# Big Science as Strategic Infrastructure: European Coordination, Public Value and Socio-economic Evidence

**DOI:** 10.1007/s41781-026-00193-4

**Published:** 2026-11-06

**Authors:** Riccardo Crescenzi, Charlotte Glass, Patricia Postigo McLaughlin, José Luis Martínez Peña

**Affiliations:** 1 Department of Geography and Environment, London School of Economics and Political Science, London, UK; 2 UK Research and Innovation (UKRI), Science and Technology Facilities Council (STFC), Swindon, UK; 3 Directorate-General for Research and Innovation (DG RTD), European Commission, Brussels, Belgium; 4 European Strategy Forum on Research Infrastructures, Brussels, Belgium; 5 Instituto de Ciencia de Materiales de Madrid (CSIC), Madrid, Spain

**Keywords:** Big Science, Research infrastructures, Technology infrastructures, Socio-economic impact, Innovation policy, Public procurement, Evaluation

## Abstract

Big Science investment is justified primarily by its contribution to scientific discovery. Yet, the political economy around public investment in Big Science has changed. Public budgets are tighter, scientific equipment is more costly, energy and environmental constraints are more visible, and geopolitical fragmentation has made technological capability a more explicit policy concern. This article examines how socio-economic evidence can support strategic choices without reducing science to short-term accounting. It argues that the economic case for public investment in Big Science, mediated through research infrastructures (RIs), should be framed around capability formation, excellent science, European coordination and system-wide impacts. Three shifts are central: from national return to European value added, from facility-level assessment to portfolio-level strategy, and from one-off impact studies to life-cycle evaluation. Better evidence should be cumulative, analytically robust and decision-relevant. It should show not only what research infrastructures produce, but also what they enable across scientific communities, firms, skills systems, regions and policy domains.

## Introduction: Why Evidence Now Matters More

The case for public investment in Big Science must begin with science itself. Big Science depends on the specific type of research infrastructures (RIs) that open new experimental possibilities and enable discoveries and research otherwise unattainable. At the same time, public investment requires a convincing account of public value. Especially (although not exclusively) in the case of Big Science, governments are asked to support facilities whose costs are large, whose planning horizons are long, and whose transformative effects may appear only after many years. Ministers face relatively short fiscal and electoral cycles. They need credible evidence that investment in RIs can also generate wider benefits for citizens, firms and territories. The analytical challenge is not to justify science only in economic terms. It is to build an economic case that is rigorous, proportionate and faithful to the public-good character of discovery-led research.

This article approaches this challenge from the perspective of Big Science in Europe. Europe is a particularly revealing case because the geography of funding and the geography of benefits rarely coincide. RIs enabling Big Science are often financed through contributions funded by national budgets, but their returns diffuse through transnational user communities, procurement networks, data systems and mobile human capital that overcome national boundaries and even those of the consortia formed to fund them. This creates the central political–economy problem addressed in this article: how to build socio-economic evidence for collective investments whose value is strategic, long-term, systemic and unevenly distributed across territories. This framing sets up the argument that follows: the principal weakness of current socio-economic evidence is not simply a shortage of impact studies, but the absence of a cumulative framework connecting facility-level evidence to national and European portfolio decisions across the full research-infrastructure life cycle.

## More Demand, Fewer Means: The New Political–Economic Moment

The renewed attention to the economics of Big Science reflects a structural change in innovation. Modern innovation is increasingly science-based, cumulative and collective. Patent evidence shows that inventions closer to scientific knowledge tend to be more valuable, while high-impact science and technology increasingly depend on teams rather than lone inventors [1, 8, 24]. New ideas require specialised human capital, complex equipment and dense interactions between scientific and technological domains.

This generates a paradox. Science has become more important for innovation [18], but large firms appear increasingly less willing to produce open corporate science internally ([2, 3]; see also [23]). Corporate research and development (R&D) continues, but the scientific orientation of corporate laboratories has weakened, leaving firms more dependent on knowledge produced in public and semi-public systems (see evidence in [2]). At the same time, new ideas are becoming harder to find, requiring more researchers, larger teams, longer training and more sophisticated instrumentation [4, 17].

The result is rising demand for public and collective investment precisely when Big Science faces more constraints. Financial capital is scarce; human capital must be created and recombined; and environmental and resource constraints are no longer peripheral, because large facilities consume resources and must increasingly demonstrate environmental responsibility.

The Draghi report placed this issue at the centre of the European Union (EU) competitiveness debate, framing research and innovation as central to productivity, competitiveness and strategic autonomy, and emphasising the role of costly equipment and political-capital coordination around large-scale innovation [9]. The Letta report added a complementary Single Market argument: Europe needs a fifth freedom around research, innovation, knowledge and education, including stronger research and technology infrastructures, if integration is to support future prosperity rather than only remove inherited market barriers [19].

This discussion is also shaped by geopolitics. Big Science has often emerged at moments of political transformation, from the Manhattan Project and Apollo to ITER and CERN. The contemporary context is different, but investments in the RIs that support Big Science are increasingly linked to technological sovereignty, industrial competitiveness, data governance and supply-chain resilience. Public support for science remains significant. Recent evidence suggests that there is no generalised collapse of trust in science; scientists continue to rank among the more trusted professional groups, and large international surveys point to moderately high levels of trust ([5], see also [20]). Yet, this aggregate picture masks important fractures: trust varies across countries, social groups and political orientations; it is more fragile on contested issues; and credible scientific evidence increasingly competes with misinformation, social media and alternative sources of authority [12, 22]. Broad support, however, does not automatically translate into political support. Indeed, the relevant challenge for Big Science may be increasing competition for priorities: even where science remains broadly trusted, it must still persuade policymakers and publics that long-term, capital-intensive investments are justified in the face of fiscal constraints, competing priorities and uneven territorial returns. That translation requires credible evidence to demonstrate value vis-à-vis alternative uses of the same scarce resources and a convincing institutional architecture.

## The European Architecture: When Variable Geometry Needs Evidence

The European architecture illustrates this political–economy problem. It shows how collective choices about RIs are made when scientific value, national accountability and European public value do not automatically align.

This problem has deep institutional roots. The Single European Act provided the European Community with an explicit treaty basis for research and technological development. This included strengthening the scientific and technological basis of European industry, complementing earlier forms of cooperation (including those developed under Euratom) and encouraging cooperation among firms, research centres and universities [14]. This legal foundation helped make possible a European RI policy alongside established intergovernmental organisations such as CERN, the European Southern Observatory (ESO), the European Synchrotron Radiation Facility (ESRF) and the European Molecular Biology Laboratory (EMBL). The historical point remains important: European scientific cooperation did not simply follow the European project; large-scale scientific collaboration was one of the ways in which Europe was built before, alongside and beyond the formal institutions of the Union. An early example of large-scale scientific cooperation within the European Communities was the Joint European Torus (JET), established as a Euratom Joint Undertaking in 1978, a quintessential Big Science project.

For several decades, Europe has managed this tension through a relatively light but consequential architecture of coordination of RIs. Transnational access, joint technology development and roadmapping have allowed Europe to pool expertise, reduce duplication and widen access without fully centralising decisions. Transnational access is especially important because it creates the functional equivalent of a single market for RI users: an Erasmus-style system through which excellent researchers can access world-leading facilities even when their own country does not host them. Joint technology development allows facilities to pool expertise around common designs. Roadmapping and EU funds support preparation of RI projects throughout their life cycles. Preparatory project funding is particularly important because it provides the necessary support for communities to carry out an implementable project.

This architecture rests on variable geometry. Not every country can or should finance every RI; instead, countries contribute selectively according to their scientific priorities, fiscal capacities and strategic interests. This makes cooperation possible without full centralisation, but it also makes evidence more important. The case for a facility must speak not only to scientific excellence, but also to how national contributions generate direct returns and European public value. The European Strategy Forum on Research Infrastructures (ESFRI) and its Roadmap provide one institutional expression of this logic. First published in 2006 and updated periodically, the Roadmap had by the 2021 edition expanded to 63 RIs of pan-European relevance [15], with the 2026 update underway and scheduled for launch in Rome in December 2026 [16]. Its importance lies less in the list itself than in the discipline it creates: landscape analysis, prioritisation and monitoring. These are the mechanisms through which Europe asks which infrastructures are needed, how they complement one another, when they require a major upgrade and when continuation should be reconsidered. In addition, several EU Member States also maintain their own strategies and roadmaps.^1^

The stakes are increasing. Most RI financing still comes from Member States, with EU-level funding historically focussed on concept and design stages, project preparation, access and coordination. Under the Commission’s proposal for the next Framework Programme, EU-level funding could contribute up to 20% of the construction costs of certain critical new research and technology infrastructures. If adopted, this would represent a significant institutional development [13]: the EU would move closer to sharing capital costs, not merely enabling coordination around them. That shift would make evidence more consequential. Decisions would need to justify why a particular investment should be supported at European level and how it complements existing capabilities at the EU level.

The 2025 European Strategy on Research and Technology Infrastructures reinforces this shift [10, 11]. The strategy places RIs and technology infrastructures (TIs) within a single innovation continuum, linking frontier science to testing, validation, scale-up and deployment. It also connects them explicitly to industrial competitiveness, skills, technological sovereignty and access for firms, including start-ups and small and medium-sized enterprises. This reframes RIs within Europe’s broader competitiveness agenda. The opportunity is that RIs become recognised as strategic assets for scientific and technological capacity. The risk is that they are judged too narrowly through short-term competitiveness metrics. The central policy challenge is therefore to build evidence and governance arrangements that can support strategic choices while preserving the scientific openness, long time horizons and collaborative logic that make RIs valuable in the first place.

This raises the question taken up next: through which impact channels do RIs deliver public value beyond the facility itself?

## Big Science as a System of Impacts

Big Science should be understood as a system, not as a set of isolated facilities. RIs connect investments, users, suppliers, national laboratories, universities, international subscriptions, firms, public agencies and wider societies. Their benefits are rarely confined to the facility itself or to the locality that hosts it. They are realised across wider networks of users, suppliers, funders, national partners and international organisations. At the same time, these impacts do not arise from RIs alone; they depend on complementary investments, capabilities and activities elsewhere in the research and innovation system. This is why impact should be mapped at the level of the capabilities and relationships that RIs help to organise, rather than only at the level of the facility itself.

The impact pathways are multiple. RIs produce publications, data and methods; create industrial benefits through procurement, supplier learning and co-development; train scientists, engineers, technicians and managers; generate new data architectures and computing capabilities; and produce cultural and educational benefits. They also create environmental costs and opportunities, because technologies developed to operate large facilities may later improve efficiency in other sectors. Their contributions therefore extend beyond research excellence to encompass innovation, skills development, environmental sustainability, science diplomacy and wider societal value.

Impact pathways are complex. Tracing and evidencing impact requires looking beyond the immediate beneficiaries or recipients. Procurement is a particularly relevant example in this regard: it creates visible interfaces between science and the economy. But procurement impacts should not be reduced to contract values or jobs inside supplier firms. Research infrastructure procurement can generate effects beyond firm boundaries through backward linkages, local supply chains, labour mobility and specialised know-how. Research by Crescenzi and Piazza on the procurement of superconducting radio-frequency cavities for the European X-ray Free Electron Laser (XFEL) illustrates this wider geography of impact. By 2017, the European XFEL contract had generated approximately 30 direct jobs within the supplier, E. Zanon, and supported an estimated 445 additional manufacturing jobs outside the firm in the municipality of Schio, Italy, compared with a synthetic counterfactual in which the contract had not been awarded [6, 7]. The word ‘additional’ is essential. Without a credible counterfactual, impact assessment can describe association but not additionality. This matters especially in regions facing industrial decline or economic shocks, where RI procurement may sustain capabilities that would otherwise have eroded. This is also why the geography of impact matters. Research infrastructures generate effects where they are located, but also where procurement contracts, supplier networks, trained people and technical knowledge land. The economic footprint of Big Science therefore has a networked geography rather than a simple host-region geography.^2^

## What Good Evidence Should Look Like

A coherent evidence framework should operate at three interconnected levels. At facility level, it should identify the scientific, technological and societal capabilities enabled by an RI. At national level, it should assess complementarity with existing investments, skills and strategic priorities. At European level, it should examine the added value created through coordination, transnational access, specialisation and the wider distribution of benefits. Evaluation across the RI life cycle should cut across all three levels.

If impacts are systemic, the evaluation problem is not whether an individual RI has impact, but how to evidence and assess the impact of different facilities and portfolios in ways that are credible, proportionate and useful for decision-making. A synchrotron, a neutron source, a social survey, a high-performance computing facility and a particle accelerator have different missions, users, technologies, geographies and time horizons. No single metric can capture their value.

At the same time, decision-makers increasingly ask portfolio-level questions: how facilities complement each other, which capabilities are missing, whether duplication is inefficient or redundancy is justified, and how new construction should be balanced against maintenance and renewal. The UK National Audit Office (NAO) made this concern explicit in March 2026, arguing that the Department for Science, Innovation and Technology and UK Research and Innovation should manage research infrastructure more effectively as a portfolio [21].

This creates a tension between diversity and coherence. Evaluation frameworks need enough common structure to support learning across facilities, but enough flexibility to respect different missions, operating models and life cycles. Coherence does not mean aggregation. It should not imply ranking all facilities on a single score or collapsing diverse benefits into one monetised number. A coherent framework should instead make impact pathways, assumptions, data sources, uncertainties and decision contexts explicit, seeking standardisation only where appropriate.

This direction is reflected in the UK Science and Technology Facilities Council (STFC) approach: applying mixed-methods, theory-based evaluation approaches whilst seeking greater consistency across facility-level evaluations and working towards providing a stronger portfolio perspective. Such an approach links facility-specific evidence to system-level questions while avoiding the false precision of one-size-fits-all assessment.

Evaluation must also follow the RI life cycle. Developing a research infrastructure is not a one-off decision that ends once construction is approved. It has a life cycle: idea, concept, design, implementation, operation, significant upgrade, and in some cases decommissioning or transition to a new phase. Preparatory evaluation should assess scientific need, users, governance, financial sustainability and expected impact pathways. Construction-phase evaluation should capture supplier learning, skills and industrial capability. Operational evaluation should examine scientific output, access, user benefits, training, data services and wider socio-economic effects. Upgrade decisions require evidence on frontier relevance and opportunity cost.

The timing of evaluation is political as well as technical. Evidence is produced within funding cycles, budget negotiations and institutional pressures. Governments may ask for evidence of impact long before the impacts  have materialised or could reasonably be expected to do so. Facilities may face reporting obligations that generate many indicators but limited insight. Funders may ask for monetised benefits even where the most important effects are uncertain, distributed or not readily quantifiable. The deeper point is that evaluation is itself part of the political economy of science. Why evaluation is undertaken, when it is undertaken, which types of impacts are considered important, what defines “good” evidence, and what counts as enough data are all judgement-laden questions. The European Commission does not currently rely on a single official impact assessment method for RIs, partly because the diversity of facilities makes a one-size-fits-all method inappropriate. At the same time, user metrics can be challenging. Even tracking which EU-funded researchers used which research infrastructures requires a long-term framework and technical expertise (experiments vary within a facility and across facilities, and  extracting meaning requires comparison with broader use data at national level and historical data). User data for each EU framework programme are only fully available a few years after its conclusion, due to grants signed in its last years and to projects being extended when a facility needs repairs. Better tools, including artificial intelligence and machine learning, may help extract information from publications, procurement records and user databases, but they cannot substitute for conceptual clarity. Human expertise and experience remain essential to shape these metrics.

A credible evidence framework therefore requires baseline and counterfactual evidence; common core data alongside facility-specific indicators; geographical and distributional analysis; evaluation across the life cycle; and clear links to actual investment decisions.

## Recommendations and Knowledge Gaps

Five implications follow for policy and practice.

First, evidence of wider socio-economic impacts should complement, not replace, scientific excellence. The purpose of evaluation is not to understand RIs through a conventional industrial lens, but to explain how scientific investments generate wider public value.

Second, the evaluation and investment strategy should recognise the value of specialised services and technological capabilities enabled by RIs, even when these are peripheral to their core scientific mission. Capabilities such as radiation-hardness testing for space technologies or the production of medical radioisotopes may depend on accelerator, reactor or laboratory capacities that cannot be readily replicated elsewhere. Their value therefore extends beyond direct service provision: they can support technological sovereignty, resilience and mission-critical industrial capabilities. These functions should be identified explicitly in investment cases, portfolio strategies and evaluation frameworks.

Third, funders should promote cumulative learning and insights by requiring evaluations to contribute to a minimum shared evidence base across RIs: baseline data, procurement tracking, user pathways, skills movement, environmental metrics and periodic follow-up. Whilst one-off studies remain necessary and useful, their value for informing strategic and portfolio-level decision-making is constrained unless they are underpinned by a degree of consistency in approach, metrics and methodologies.

Fourth, portfolio-level strategy should be made more explicit nationally and at European level. Roadmaps and investment cases should include clear portfolio tests: complementarity, duplication, capability gaps, upgrade needs and the expected distribution of benefits.

Fifth, impact evaluation should be designed into RIs from the start. This means identifying impact pathways early, identifying relevant and meaningful indicators, collecting baseline data, tracking project, procurement and user networks, and planning evaluation points across the life cycle. Retrofitting evidence collection and analysis after major decisions have been taken is costly and often methodologically weak.

The main research gaps concern interaction, distribution and additionality. We need to know more about how impact channels reinforce one another; how procurement effects diffuse across firms, supply chains and places; how skills move between infrastructures and industry; how environmental costs and benefits can be evaluated consistently; and how European coordination changes the distribution of benefits. We also need better methods to identify and value the strategic capabilities and option value created by RIs, including services outside their core scientific mission that may become critical in response to future technological, industrial or societal needs. Above all, the field needs more counterfactual evidence. Policymakers need to know not only what Big Science produces, but what would be lost without it.

Big Science will continue to require political capital. The task for economists, evaluators and policymakers is to ensure that this political capital is supported by evidence that is rigorous without being reductive, strategic without being technocratic, and attentive to the long-term public value of science.

## Data Availability

No datasets were generated or analysed during the current study.

## Footnotes

1 https://www.esfri.eu/national-roadmaps

2 Data from the ESFRI Roadmap 2026 indicate that 64% of the leadership of listed research infrastructures is concentrated in five EU Member States and Associated Countries. Against this concentration of institutional leadership, procurement may provide an important channel through which the economic benefits of research infrastructure investment diffuse more widely across firms and territories.
